# Supplementary material for: When everything changes at once: finding a new normal after genome duplication
Source: Proc Biol Sci. 2020 Nov 18;287(1939):20202154. doi: 10.1098/rspb.2020.2154 (PMC7739491; doi:10.1098/rspb.2020.2154)
Supplement: Table S1 [file rspb20202154supp1.pdf]

Trait Class	Trait	Rel to 2X	Species where reported	Notes	Ploidy	Type	Source	Ref
**Cell size**	**Cell size**	**Larger**	*Arabidopsis thaliana*	Differs by cell type	4X (6X,8X)	Neo	Colch, Nat	(1-4)
			Atriplex confertifolia	Differs by cell type	4X, 8X, 10X	Est	Natural	(5)
			Camellia sinensis		4X	Neo	Cultivar	(6)
			Citrus limona		4X	Neo	Natural	(7)
			Convolvulus pluricaulis		4X	Est	Natural	(8)
			Festuca arundinaceae		4X,6X,8X,10X	Est	Natural	(9)
			Isatis indigotica		4X	Neo	Colchicine	(10)
			Lolium perenne, multiflorum		4X	Culv		(11)
			Phlox amabilis		4X,6X	Est	Natural	(12)
			Poncirus trifoliata		4X	Neo	Natural	(13)
			Salix viminalis		4X	Neo	Colchicine	(14)
			Solanum phurela		4X	Neo	Complex	(15)
	**Guard cell size**	**Larger**	*Arabidopsis thaliana*		4X	Neo	Colchicine	(2)
			Asparagus officinalis		4X	Neo	Colchicine	(16)
			Brassica rapa (Pak choi)		4X	Neo	Colchicine	(17)
			Chamerion angustofolium	Neo > Est	4X	Est,Neo	Nat, Colch	(18)
			Citrus limona		4X	Neo	Natural	(7)
			Citrus reticulata		4X	Neo	Natural	(19)
			Clematis heracleifolia		4X	Neo	Colchicine	(20)
			Cynodon dactylon		4X	Est	Natural	(21)
			Festuca arundinaceae		4X,6X,8X,10X	Est	Natural	(9)
			Fragaria vesca		4X	Neo	Colchicine	(22)
			Jatropha curcas		4X,8X	Neo	Colchicine	(23)
			Nigella sativa		4X	Neo	Colchicine	(24)
			Ocimum kilimandscharicum		4X	Neo	Colchicine	(25)
			Phlox amabilis		4X,6X	Est	Natural	(12)
			Plantago media	neo slightly > than est	4X	Neo,Est	Nat, Colch	(26)
			Plumbago auricalata		4X	Neo	Pendimethalin	(27)
			Poncirus trifoliata		4X	Neo	Natural	(13, 19)
			Populus tremuloides		4X	Est	Natural	(28)
			Raphanus sativus		4X	Neo	Colchicine	(29)
			Spathiphylum walisii		4X	Neo	Colchicine	(30)
			Vanilla planifolia		4X	Neo	Natural	(31)
			Ziziphus jujuba		4X	Neo	Colchicine	(32)
		**No diff.**	*Solanum* spp.		4X	Neo	Oryzalin	(33)
	**Pollen**	**Larger**	*Brassica rapa* (Pak choi)		4X	Neo	Colchicine	(17)

	**grains**		*Chamerion angustofolium*		4X	Est	Natural	(34)
			Jatropha curcas		4X,8X	Neo	Colchicine	(23)
			Plantago media	Neo > Est	4X	Neo,Est	Colchicine	(26)
			Ziziphus jujuba		4X	Neo	Colchicine	(32)
**Gas Exchange**	**Stomatal density**	**Lower**	*Asparagus officinalis*		4X	Neo	Colchicine	(16)
			Betula papyrifera		4X	Est	Natural	(35)
			Camellia sinensis		4X	Neo	Cultivar	(6)
			Chamerion angustofolium		4X	Est,Neo	Nat, Colch	(18)
			Citrus limona		4X	Neo	Natural	(7)
			Citrus reticulata		4X	Neo	Natural	(19)
			Clematis heracleifolia		4X	Neo	Colchicine	(20)
			Convovulus pluricaulis		4X	Est	Natural	(8)
			Cynodon dactylon		4X	Est	Natural	(21)
			Festuca arundinaceae		4X,6X,8X,10X	Est	Natural	(9)
			Fragaria vesca		4X	Neo	Colchicine	(22)
			Jatropha curcas		4X,8X	Neo	Colchicine	(23)
			Ocimum kilimandscharicum		4X	Neo	Colchicine	(25)
			Phlox amabilis		4X,6X	Est	Natural	(12)
			Plumbago auricalata		4X	Neo	Pendimethalin	(27)
			Poncirus trifoliata		4X	Neo	Natural	(13, 19)
			Spathiphylum walisii		4X	Neo	Colchicine	(30)
			Ziziphus jujuba		4X	Neo	Colchicine	(32)
		**No diff.**	*Solanum* spp.		4X	Neo	Oryzalin	(33)
	**Stomatal Conduct.**	**Higher**	*Arabidopsis thaliana*	Genotypic variation	4X	Neo	Colch/Nat	(36)
			Arabidopsis thaliana	Altered ROS/ABA?	4X	Neo	Colchicine	(4)
			Citrus limona	Only when stressed	4Xroot	Neo	Natural	(37)
			Cochlearia officinalis		4X	Est	Natural	(38)
			Atriplex canescens		4X,6X	Est	Natural	(39)
			Festuca arundinaceae		4X,6X,8X,10X	Est	Natural	(9)
			Lonicera japonica	In drought only	4X	Neo	Colchicine	(40)
			Populus tremuloides		3X	Est	Natural	(28)
			Phlox drumondii	Only in 11th gen Colchi	4X	Neo	Colch(C_0,C_{11})	(41)
			Robinia pseudoacacia	In salinity	4X	Est	Natural	(42)
			Solidago gigantea		4X	Est	Natural	(43)
			Salix viminalis		4X	Neo	Colchicine	(14)
			Spathiphylum walisii	In drought	4X	Neo	Colchicine	(30)
		**Lower**	*Camellia sinensis*		4X	Neo	Cultivar	(6)
			Citrus limona	Unstressed; Higher ABA	4Xroot	Neo	Natural	(37)

			Jatropha curcas		4X,8X	Neo	Colchicine	(23)
		**No diff**	*Medicago sativa*		4X,8X	Neo	Colchicine	(44)
			Solanum bulbocastanum		4X	Neo	Oryzalin	(45)
**Photo-synthesis**	**Photosynth rate**	**Higher**	*Atriplex canescens*		4X,6X	Est	Natural	(39)
			Atriplex confertifolia		4X, 8X, 10X	Est	Natural	(5)
			Brassica rapa (Pak choi)		4X	Neo	Colchicine	(17)
			Cochlearia officinalis		4X	Est	Natural	(38)
			Isatis indigotica		4X	Neo	Colchicine	(10)
			Jasione maritima		4X	Neo	Colchicine	(46)
			Lagenaria sphaerica		4X	Neo	Colchicine	(47)
			Lonicera japonica	In drought only	4X	Neo		(40)
			Manihot esculenta		4X	Neo	Colchicine	(48, 49)
			Oryza sativa		4X	Neo	Colchicine	(50)
			Phlox drumondii		4X	Neo	Colch(C_0,C_{11})	(41)
			Salix viminalis		4X	Neo	Colchicine	(14)
			Ziziphus jujuba		4X	Neo	Colchicine	(32)
		**Lower**	*Jatropha curcas*		8X	Neo	Colchicine	(23)
			Phlox drummondii		4X	Neo	Colchicine	(51)
		**No diff**	*Arabidopsis thaliana*	Genotypic variation	4X	Neo	Colch/Nat	(52)
			Jatropha curcas		4X	Neo	Colchicine	(23)
			Solidago gigantea		4X	Est	Natural	(43)
	**Chlorophyll content**	**Higher**	*Asparagus officinalis*		4X	Neo	Colchicine	(16)
			Atriplex confertifolia	C4, Bundle sheath only	4X, 8X, 10X	Est	Natural	(5)
			Brassica rapa (Pak choi)		4X	Neo	Colchicine	(17)
			Coccinia palmate		4X	Neo	Colchicine	(47)
			Dendranthema nankingense		4X	Neo	Colchicine	(53)
			Lagenaria sphaerica		4X	Neo	Colchicine	(47)
			Manihot esculenta		4X	Neo	Colchicine	(49)
			Nigella sativa		4X	Neo	Colchicine	(24)
			Oryza sativa		4X	Neo	Colchicine	(50)
			Pauwlonia tomentosa		4X	Neo	Colchicine	(54)
			Salix viminalis		4X	Neo	Colchicine	(14)
		**No diff**	*Anemone sylvestris*		4X	Neo	Oryzalin	(55)
			Jatropha curcas		4X,8X	Neo	Colchicine	(23)
			Medicago sativa		4X,8X	Neo	Colchicine	(44)
			Solanum bulbocastanum		4X	Neo	Oryzalin	(45)
	**Leaf color**	**Darker**	*Asparagus officinalis*		4X	Neo	Colchicine	(16)
			Capsicum annuum		4X	Neo	Colchicine	(56)

			Citrus limona		4X	Neo	Natural	(7)
			Nigella sativa		4X	Neo	Colchicine	(24)
			Ziziphus jujuba		4X	Neo	Colchicine	(32)
**Stress tolerance**	**Drought tolerance**	**Higher**	*Arabidopsis thaliana*	Altered ROS/ABA?	4X	Neo	Colchicine	(4)
			Atriplex canescens		4X,6X	Est	Natural	(39, 57)
			Betula spp.	Altered pit structure	4X	Est	Natural	(58)
			Coccinia palmate		4X	Neo	Colchicine	(47)
			Chamerion angustifolium	(established tet only)	4X	Neo, Est	Colch,Nat	(18)
			Citrus limona	Higher ABA	4Xroot	Neo	Natural	(37)
			Dendranthema nankingense		4X	Neo	Colchicine	(53)
			Hordeum marinum		4X	Est	Natural	(59)
			Lagenaria sphaerica		4X	Neo	Colchicine	(47)
			Lonicera japonica		4X	Neo		(40)
			Lycium ruthenicum	4X has high ABA	4X	Neo	Colchicine	(60)
			Malus x domestica		4X	Neo	Colchicine	(61)
			Nicotiana benthamiana		8X (4X)	Neo	Colchicine	(62)
			Poncirus trifoliata		4X	Neo	Natural	(13)
			Robinia pseudoacacia	K^+/Na^+ rem. High in 4X	4X	Est	Natural	(42)
			Spathiphylum wallisii		4X	Neo	Colchicine	(30)
	**Salt tolerance**	**Higher**	*Arabidopsis thaliana*	Altered ROS/ABA?	4X	Neo	Colchicine	(4)
			Arabidopsis thaliana	Higher K^+; K^+/Na^+ ratio	4X	Neo	Colch,Nat	(63)
			Beta vulgaris	Higher K^+ & K^+/Na^+ ratio	4X	Neo	Cultivars	(64)
			Brassica rapa (Turnip)	Higher K^+/Na^+ ratio	4X	Neo		(65)
			Dendranthema nankingense		4X	Neo	Colchicine	(53)
			Malus x domestica		4X	Neo	Colchicine	(66)
			Manihot esculenta		4X	Neo	Colchicine	(48)
			Oryza sativa	Protective gap in roots	4X	Neo	Colchicine	(67)
			Robinia pseudoacacia		4X	Est	Natural	(68)
			Solanum lycopersicum		4X	Neo	Colchicine	(69)
		**Lower**	*Chloris gayana*		4X	Cultivar		(70)
			Cochlearia officinalis		4X	Est	Natural	(38)
		**Variab.**	*Citrus deliciosa*	Var. by watering regime	4X	Neo	Natural	(71)
			Poncirus trifoliata	Var. by watering regime	4X	Neo	Natural	(71)
	**Hydric**	**Lower**	*Phlox drummondii*		4X	Est	Natural	(72)
	**Cold**	**Higher**	*Dendranthema nankingense*		4X	Neo	Colchicine	(53)
			Plumbago auricalata		4X	Neo	Pendimethalin	(27)
		**Lower**	*Festuca pratensis*		4X	Est	Natural	(73)
			Lolium perenne		4X	Cultivars		(74)

	**Freezing**	**Lower**	*Chamerion angustifolium*		4X	Est	Natural	(75)
			Solidago canadensis		4X	Est	Natural	(76)
	**Heat**	**Higher**	*Asparagus officinalis*		4X	Neo	Colchicine	(16)
			Dioscorea zingiberensis		4X	Neo	Colchicine	(77)
			Lonicera japonica	In drought only	4X	Neo	Colchicine	(78)
		**Lower**	*Dendranthema nankingense*		4X	Neo	Colchicine	(53)
		**No diff**	*Fragaria vesca*		4X	Neo	Colchicine	(22)
	**UV tol.**	**Higher**	3 conifer spp in Japan		4X	Est	Natural	(79)
	**ROS scavenging**	**Higher**	*Anemone sylvestris*		4X	Neo	Oryzalin	(55)
			Cenchrus spp.		4X	Est	Natural	(80)
			Nicotiana benthamiana		8X (4X)	Neo	Colchicine	(62)
			3 conifer spp in Japan		4X	Est	Natural	(79)
	**Anti-ox activity**	**Higher**	*Anemone sylvestris*		4X	Neo	Oryzalin	(55)
			Brassica rapa (Turnip)		4X	Neo		(65)
			Dendranthema nankingense		4X	Neo	Colchicine	(53)
			Dioscorea zingiberensis		4X	Neo	Colchicine	(77)
			Nicotiana benthamiana		8X (4X)	Neo	Colchicine	(62)
**Hydraulics**	**Xylem diameter**	**No diff**	*Solanum* spp		4X	Neo	Oryzalin	(33)
		**Higher**	*Atriplex canescens*		4X	Est	Natural	(57)
			Betula spp.		4X	Est	Natural	(58)
			Capsicum annuum		4X	Neo	Colchicine	(56)
			Chamerion angustifolium		4X	Neo, Est	Colch,Nat	(18)
	**Hydraulic cond**	**Lower**	*Atriplex canescens*		4X, 6X	Est	Natural	(39),(57)
		**Higher**	*Ocimum kilimandscharicum*		4X	Neo	Colch (C_2)	(25)
			Chamerion angustifolium	Est >> neo	4X	Neo, Est	Colch,Nat	(18)
	**Cavitation Resistance**	**Higher**	*Betula* spp.	Altered pit structure	4X	Est	Natural	(58)
		**No diff**	*Atriplex canescens*		4X	Est	Natural	(57)
			Chamerion angustifolium		4X	Neo, Est	Colch,Nat	(18)
	**Vasc.Bund.#**	**Higher**	*Plumbago auricalata*		4X	Neo	Pendimethalin	(27)
	**WUE**	**Higher**	*Atriplex canescens*		4X, 6X	Est	Natural	(39)
			Populus tremuloides		3X	Est	Natural	(28)
**Devel.**	**Growth rate**	**Lower**	*Arabidopsis thaliana*		4X, 6X, 8X	Neo	Colchicine	(1, 2)
			Citrus limona		4X	Neo	Natural	(7)
			Cochlearia officinalis	Est > Neo	4X,8X	Neo,Est	Colch,Nat	(81)
			Lycium ruthenicum		4X	Neo	Colchicine	(60)
			Nicotiana benthamiana		8X (4X)	Neo	Colchicine	(62)
			Phlox drummondii		4X	Est	Natural	(72)
			Poncirus trifoliata		4X	Neo	Natural	(13)

		Salix viminalis		4X	Neo	Colchicine	(14)
		Ziziphus jujuba		4X	Neo	Colchicine	(32)
	**No diff**	*Fragaria vesca*		4X	Neo	Colchicine	(22)
**Root system**	**Larger**	*Arabidopsis thaliana*		4X	Neo	Colchicine	(1, 2, 4)
		Capsicum annuum		4X	Neo	Colchicine	(56)
		Salix viminalis		4X	Neo	Colchicine	(14)
	**Thicker**	*Citrus limona*		4X	Neo	Natural	(7)
		Plumbago auricalata		4X	Neo	Pendimethalin	(27)
**Flowering**	**Delayed**	*Arabidopsis thaliana*		4X, (6X, 8X)	Neo	Colchicine	(1, 4)
		Convolvulus pluricaulis		4X	Est	Natural	(8)
		Nigella sativa		4X	Neo	Colchicine	(24)
		Phlox drummondii		4X	Est	Natural	(72)
**Flower Organs**	**Larger**	*Anemone sylvestris*		4X	Neo	Oryzalin	(55)
		Arabidopsis thaliana		4X	Neo	Colchicine	(2-4)
		Chamerion angustifolium		4X	Est	Natural	(34)
		Convolvulus pluricaulis		4X	Est	Natural	(8)
		Phlox amabilis		4X, 6X	Est	Natural	(12)
		Raphanus sativus		4X	Neo	Colchicine	(29)
		Ziziphus jujuba		4X	Neo	Colchicine	(32)
**Leaf thickness**	**Higher**	*Arabidopsis thaliana*		4X	Neo	Colchicine	(2)
		Capsicum annuum		4X	Neo	Colchicine	(56)
		Citrus limona		4X	Neo	Natural	(7)
		Citrus reticulata		4X	Neo	Natural	(19)
		Clematis heracleifolia		4X	Neo	Colchicine	(20)
		Convolvulus pluricaulis		4X	Est	Natural	(8)
		Isatis indigotica		4X	Neo	Colchicine	(10)
		Jatropha curcas		4X,8X	Neo	Colchicine	(23)
		Lonicera japonica		4X	Neo		(40)
		Morus alba		4X	Neo	Colchicine	(82)
		Nigella sativa		4X	Neo	Colchicine	(24)
		Pauwlonia tomentosa		4X	Neo	Colchicine	(54)
		Poncirus trifoliata	Also thick epidermis	4X	Neo	Natural	(13, 19)
		Raphanus sativus		4X	Neo	Colchicine	(29)
		Solanum phureja		4X	Neo	Complex	(15)
		Spathiphylum wallisii		4X	Neo	Colchicine	(30)
	**No diff**	*Solanum* spp		4X	Neo	Oryzalin	(33)
**Seed size/weight**	**Higher**	*Arabidopsis thaliana*		4X	Neo	Colchicine	(4)
		Oryza sativa		4X	Neo	Colchicine	(20)

			Plantago media	Neo > Est	4X	Neo, Est	Colch,Nat	(26)
			Themeda triandra		4X	Est	Natural	(83)
	**Plant Height**	**Lower**	*Citrus reticulata*		4X	Neo	Natural	(19)
			Convolvulus pluricaulis		4X	Est	Natural	(8)
			Oryza sativa		4X	Neo	Colchicine	(20)
			Poncirus trifoliata		4X	Neo	Natural	(19)
		**Higher**	*Anemone sylvestris*		4X	Neo	Oryzalin	(55)
			Capsicum annuum		4X	Neo	Colchicine	(56)
			Chamerion angustifolium	Established 4X only	4X	Neo, Est	Colch,Nat	(84)
			Morus alba		4X	Neo	Colchicine	(82)
			Nigella sativa		4X	Neo	Colchicine	(24)
			Solidago gigantea		4X	Est	Natural	(43)
		**No diff**	*Raphanus sativus*		4X	Neo	Colchicine	(29)
			Solidago gigantea		6X	Est	Natural	(43)
	**Biomass**	**Lower**	*Chamerion angustifolium*		4X	Neo, Est	Colch,Nat	(84)
			Citrus limona	Whole tree 4X	4X	Neo	Natural	(7)
			Citrus limona	4X root, 2X scion	4X	Neo	Natural	(37)
			Convolvulus pluricaulis		4X	Est	Natural	(8)
			Malus x domestica	Low IAA and BR	4X	Neo	Colchicine	(85)
			Spathiphylum wallisii	Stems shorter, thicker	4X	Neo	Colchicine	(30)
			Ziziphus jujuba		4X	Neo	Colchicine	(32)
		**Higher**	*Cochlearia officinalis*		4X,8X	Neo,Est	Colch,Nat	(81)
**Cell walls**	**Lignin/Cellul**	**Lower**	*Arabidopsis thaliana*		4X, 6X, 8X	Neo	Colchicine	(1)
	**Crushed**	**More**	*Arabidopsis thaliana*		6X, 8X	Neo	Colchicine	(1)
	**Thickness**	**Lower**	*Arabidopsis thaliana*		4X, 6X, 8X	Neo	Colchicine	(1)
**Metab.**	**Starch**	**Higher**	*Brassica rapa* (Pak choi)	Larger, more granules	4X	Neo	Colchicine	(17)
	**Sucrose**	**Higher**	*Brassica rapa* (Pak choi)		4X	Neo	Colchicine	(17)
			Poncirus trifoliata		4X	Neo	Natural	(13)
			Plantago media	neotet > established	4X	Neo, Est	Colch,Nat	(26)
			Ziziphus jujuba		4X	Neo	Colchicine	(32)
	**1° metabolism**	**Higher**	*Citrus reticulata*		4X	Neo	Natural	(19)
			Ocimum kilimandscharicum		4X	Neo	Colch (C_2)	(25)
			Poncirus trifoliata	Lower 2° metab	4X	Neo	Natural	(19, 86)

Notes:

Ref = References:

1. Corneillie S, De Storme N, Van Acker R, Fangel JU, De Bruyne M, De Rycke R, et al. Polyploidy Affects Plant Growth and Alters Cell Wall Composition. Plant Physiol. 2019;179(1):74-87.
2. Li X, Yu E, Fan C, Zhang C, Fu T, Zhou Y. Developmental, cytological and transcriptional analysis of autotetraploid Arabidopsis. Planta. 2012;236(2):579-96.
3. Robinson DO, Coate JE, Singh A, Hong L, Bush M, Doyle JJ, et al. Ploidy and Size at Multiple Scales in the Arabidopsis Sepal. Plant Cell. 2018;30(10):2308-29.
4. del Pozo JC, Ramirez-Parra E. Deciphering the molecular bases for drought tolerance in Arabidopsis autotetraploids. Plant Cell Environ. 2014;37(12):2722-37.
5. Warner DA, Edwards GE. Effects of Polyploidy on Photosynthetic Rates, Photosynthetic Enzymes, Contents of DNA, Chlorophyll, and Sizes and Numbers of Photosynthetic Cells in the C(4) Dicot Atriplex confertifolia. Plant Physiol. 1989;91(3):1143-51.
6. Ng'etich W, Wachira FN. Variations in leaf anatomy and gas exchange in tea clones with different ploidy. J Hort Sci Biotech. 2003;78:173-6.
7. Allario T, Brumos J, Colmenero-Flores JM, Tadeo F, Froelicher Y, Talon M, et al. Large changes in anatomy and physiology between diploid Rangpur lime (Citrus limonia) and its autotetraploid are not associated with large changes in leaf gene expression. J Exp Bot. 2011;62(8):2507-19.
8. Malik CP, Tandon SL. Morphological and Cytological Studies of a Natural Polyploid Complex in Convolvulus pluricaulis Chois. Cytologia. 1959;24:523-31.
9. Byrne MC, Nelson CJ, Randall DD. Ploidy effects on anatomy and gas exchange of tall fescue leaves. Plant Physiol. 1981;68(4):891-3.
10. Zhou Y, Kang L, Liao S, Pan Q, Ge X, Li Z. Transcriptomic analysis reveals differential gene expressions for cell growth and functional secondary metabolites in induced autotetraploid of Chinese woad (Isatis indigotica Fort.). PLoS One. 2015;10(3):e0116392.
11. Sugiyama S. Polyploidy and cellular mechanisms changing leaf size: comparison of diploid and autotetraploid populations in two species of Lolium. Ann Bot. 2005;96(5):931-8.
12. Chansler MT, Ferguson CJ, Fehlberg SD, Prather LA. The role of polyploidy in shaping morphological diversity in natural populations of Phlox amabilis. Am J Bot. 2016;103(9):1546-58.
13. Wei T, Wang Y, Xie Z, Guo D, Chen C, Fan Q, et al. Enhanced ROS scavenging and sugar accumulation contribute to drought tolerance of naturally occurring autotetraploids in Poncirus trifoliata. Plant Biotechnol J. 2019;17(7):1394-407.
14. Dudits D, Torok K, Cseri A, Paul K, Nagy AV, Nagy B, et al. Response of Organ Structure and Physiology to Autotetraploidization in Early Development of Energy Willow Salix viminalis. Plant Physiol. 2016;170(3):1504-23.
15. Stupar RM, Bhaskar PB, Yandell BS, Rensink WA, Hart AL, Ouyang S, et al. Phenotypic and transcriptomic changes associated with potato autopolyploidization. Genetics. 2007;176(4):2055-67.

16. Chen H, Lu Z, Wang J, Chen T, Gao J, Zheng J, et al. Induction of new tetraploid genotypes and heat tolerance assessment in *Asparagus officinalis* L. Scientia Horticulturae. 2020;264:109168.
17. Zhang C, Wang H, Xu Y, Zhang S, Wang J, Hu B, et al. Enhanced Relative Electron Transport Rate Contributes to Increased Photosynthetic Capacity in Autotetraploid Pak Choi. Plant Cell Physiol. 2020;61(4):761-74.
18. Maherali H, Walden AE, Husband BC. Genome duplication and the evolution of physiological responses to water stress. New Phytol. 2009;184(3):721-31.
19. Tan F-Q, Tu H, Wang R, Wu X-M, Xie K-D, Chen JJ, et al. Metabolic adaptation following genome doubling in citrus doubled diploids revealed by non-targeted metabolomics. Metabolomics. 2017;13:143.
20. Wu Y, Li W, Dong J, Yang N, Zhao X, Yang W. Tetraploid induction and cytogenetic characterization for *Clematis heracleifolia*. Caryologia. 2013;66:215-20.
21. Chaves ALA, Chiavegatto RB, Gavilanes ML, Benites FRG, Techio VH. Effect of polyploidy on the leaf epidermis structure of *Cynodon dactylon* (L.) Pers. (Poaceae). Biologia. 2018;73:1007-13.
22. Wei N, Du Z, Liston A, Ashman TL. Genome duplication effects on functional traits and fitness are genetic context and species dependent: studies of synthetic polyploid Fragaria. Am J Bot. 2020;107(2):262-72.
23. Niu L, YTao Y-B, Chen M-S, Fu Q, Dong Y, He H, et al. Identification and characterization of tetraploid and octoploid *Jatropha curcas* induced by colchicine. Caryologia. 2016;69:58-66.
24. Dixit V, Verma S, Chaudhary BR. Changes in ploidy and its effect on thymoquinone concentrations in *Nigella sativa* L. seeds. J Hort Sci Biotech. 2016;90:537-42.
25. Bose RB, Choudhury JK. A Comparative Study of the Cytotaxonomy, Pallynology, Physiology of 'Diploid' and 'Polyploid' Plants of *Ocimum Kilimandscharicum* Guerke and their Yield of Raw Material and Volatile Contents. Caryologia. 1962;15:435-54.
26. Van Dijkt P, Van Delden W. Evidence for autotetraploidy in *Plantago media* and comparisons between natural and artificial cytotypes concerning cell size and fertility. Heredity. 1990;65:349-57.
27. Jiang Y, Liu S, Hu J, He G, Liu YY, Chen X, et al. Polyploidization of *Plumbago auriculata* Lam. in vitro and its characterization including cold tolerance. Plant Cell, Tissue and Organ Culture. 2020;140:315-25.
28. Greer BT, Still C, Cullinan GL, Brooks JR, Meinzer FC. Polyploidy influences plant-environment interactions in quaking aspen (Populus tremuloides Michx.). Tree Physiol. 2018;38(4):630-40.
29. Cheng W, Tang M, Xie Y, Xu L, Wang Y, Luo X, et al. Transcriptome-based gene expression profiling of diploid radish (Raphanus sativus L.) and the corresponding autotetraploid. Mol Biol Rep. 2019;46(1):933-45.
30. Van Laere K, Franca SC, Vansteenkiste H, Van Huylenbroeck J, Steppe K, Van Labeke M-C. Influence of ploidy level on morphology, growth and drought
susceptibility in *Spathiphyllum wallisii*. Acta Physiol Plant. 2011;33:1149-56.

31. Bory S, Catrice O, Brown S, Leitch IJ, Gigant R, Chiroleu F, et al. Natural polyploidy in Vanilla planifolia (Orchidaceae). Genome. 2008;51(10):816-26.
32. Wang L, Luo Z, Wang L, Deng W, Wei H, Liu P, et al. Morphological, cytological and nutritional changes of autotetraploid compared to its diploid counterpart in Chinese jujube (*Ziziphus jujuba* Mill.). Scientia Horticulturae. 2019;249:263-70.
33. Aversano R, Caruso I, Aronne G, De Micco V, Scognamiglio N, Carputo D. Stochastic changes affect Solanum wild species following autopolyploidization. J Exp Bot. 2013;64(2):625-35.
34. Mosquin T. Evidence for Autopolyploidy in *Epilobium angustifolium* (Onagraceae). Evolution. 1967;21:713-9.
35. Li W-L, Berlyn GP, Ashton PMS. Polyploids and their structural and physiological characteristics relative to water deficit in *Betula papyrifera* (Betulaceae). Am J Bot. 1996;83:15-20.
36. Monda K, Araki H, Kuhara S, Ishigaki G, Akashi R, Negi J, et al. Enhanced Stomatal Conductance by a Spontaneous Arabidopsis Tetraploid, Me-0, Results from Increased Stomatal Size and Greater Stomatal Aperture. Plant Physiol. 2016;170(3):1435-44.
37. Allario T, Brumos J, Colmenero-Flores JM, Iglesias DJ, Pina JA, Navarro L, et al. Tetraploid Rangpur lime rootstock increases drought tolerance via enhanced constitutive root abscisic acid production. Plant Cell Environ. 2013;36(4):856-68.
38. Bray S, Wolf EM, Zhou M, Busoms S, Bohutinska M, Paajanen P, et al. Convergence and novelty in adaptation to whole genome duplication in three independent polyploids. BioRXiv. 2020:2020.03.31.017939.
39. Senock RS, Barrow JR, Gibbens RP, Herbel CH. Ecophysiology of the polyploid shrub *Atriplex canescens* (Chenopodiaceae) growing *in situ* in the northern Chihuahuan Desert. J Arid Environ. 1991;21:45-57.
40. Li WD, Biswas DK, Xu H, Xu CQ, Wang XZ, Liu JK, et al. Photosynthetic responses to chromosome doubling in relation to leaf anatomy in Lonicera japonica subjected to water stress. Funct Plant Biol. 2009;36(9):783-92.
41. Vyas P, Bisht MS, Miyazawa SI, Yano S, Noguchi K, Terashima I, et al. Effects of polyploidy on photosynthetic properties and anatomy in leaves of Phlox drummondii. Funct Plant Biol. 2007;34(8):673-82.
42. Wang Z, Wang M, Liu L, Meng F. Physiological and proteomic responses of diploid and tetraploid black locust (Robinia pseudoacacia L.) subjected to salt stress. Int J Mol Sci. 2013;14(10):20299-325.
43. Hull-Sanders HM, Johnson RH, Owen HA, Meyer GA. Effects of polyploidy on secondary chemistry, physiology, and performance of native and invasive genotypes of Solidago gigantea (Asteraceae). Am J Bot. 2009;96(4):762-70.
44. Pfeiffer T, Schrader LE, Bingham ET. Physiological Comparisons of Isogenic Diploid-Tetraploid, Tetraploid-Octoploid Alfalfa Populations. 1980;20:299-303.
45. Caruso I, Dal Piaz F, Malafronte N, De Tommasi N, Aversano R, Zottele CW, et al. Impact of Ploidy Change on Secondary Metabolites and Photochemical Efficiency in *Solanum Bulbocastanum*. Natural Product Communications. 2913;8:1387-92.
46. Siopa C, Dias MC, Castro M, Loureiro J, Castro S. Is selfing a reproductive assurance promoting polyploid establishment? Reduced fitness, leaky self-incompatibility and lower inbreeding depression in neotetraploids. Am J Bot. 2020;107(3):526-38.

47. Ntuli NR, Zobolo AM. Effect of water stress on growth of colchicine induced polyploid *Coccinia palmata* and *Lagenaria sphaerica* plants. African J Biotech. 2008;7:3548-652.
48. An F, Fan J, Li J, Li QX, Li K, Zhu W, et al. Comparison of leaf proteomes of cassava (Manihot esculenta Crantz) cultivar NZ199 diploid and autotetraploid genotypes. PLoS One. 2014;9(4):e85991.
49. Yin L, Qu J, Zhou H, Shang X, Fang H, Lu J, et al. Comparison of leaf transcriptomes of cassava "Xinxuan 048" diploid and autotetraploid plants. Genes Genomics. 2018;40(9):927-35.
50. Yang PM, Zhou XR, Huang QC. The mechanism of starch content increase in grain of autotetraploid rice (*Oryza sativa* L.). Photosynthetica. 2019;57:680-7.
51. Bazzaz FA, Levin DA, Levy M, Schmierbach MR. The effect of chromosome doubling on photosynthesis rates in *Phlox*. Photosynthetica. 1982;16:89-92.
52. Solhaug EM, Ihinger J, Jost M, Gamboa V, Marchant B, Bradford D, et al. Environmental Regulation of Heterosis in the Allopolyploid Arabidopsis suecica. Plant Physiol. 2016;170(4):2251-63.
53. Liu S, Chen S, Chen Y, Guan Z, Yin D, Chen F. *In vitro* induced tetraploid of *Dendranthema nankingense* (Nakai) Tzvel. shows an improved level of abiotic stress tolerance. Scientia Horticulturae. 2011;127:411-9.
54. Yan L, Fan G, Deng M, Zhao Z, Dong Y, Li Y. Comparative proteomic analysis of autotetraploid and diploid Paulownia tomentosa reveals proteins associated with superior photosynthetic characteristics and stress adaptability in autotetraploid Paulownia. Physiol Mol Biol Plants. 2017;23(3):605-17.
55. Zahumenická P, Fernández E, Šedivá J, Žiarovská J, Ros-Santaella JL, Martinez-Fernandez D, et al. Morphological, physiological and genomic comparisons between diploids and induced tetraploids in Anemone sylvestris L. Plant Cell Tiss Organ Cult 2018;132:317-27.
56. Kulkarni M, Borse T. Induced polyploidy with gigas expression for root traits in *Capsicum annuum* (L.). Plant Breeding. 2010;129:461-4.
57. Hao GY, Lucero ME, Sanderson SC, Zacharias EH, Holbrook NM. Polyploidy enhances the occupation of heterogeneous environments through hydraulic related trade-offs in Atriplex canescens (Chenopodiaceae). New Phytol. 2013;197(3):970-8.
58. Zhang WW, Song J, Wang M, Liu YY, Li N, Zhang YJ, et al. Divergences in hydraulic architecture form an important basis for niche differentiation between diploid and polyploid Betula species in NE China. Tree Physiol. 2017;37(5):604-16.
59. Zhou K, Liu B, Wang Y, Zhang X, Sun G. Evolutionary mechanism of genome duplication enhancing natural autotetraploid sea barley adaptability to drought stress. Environmental and Experimental Botany. 2019;159:44-54.
60. Rao S, Tian Y, Xia X, Li Y, Chen J. Chromosome doubling mediates superior drought tolerance in Lycium ruthenicum via abscisic acid signaling. Hortic Res. 2020;7:40.
61. Zhang F, Xue H, Lu X, Zhang B, Wang F, Ma Y, et al. Autotetraploidization enhances drought stress tolerance in two apple cultivars. Trees. 2015;29:1773-80.

62. Deng B, Du W, Liu C, Sun W, Tian S, Dong H. Antioxidant response to drought, cold and nutrient stress in two ploidy levels of tobacco plants: low resource requirement confers polytolerance in polyploids? Plant Growth Regulation. 2012;66:37-47.
63. Chao DY, Dilkes B, Luo H, Douglas A, Yakubova E, Lahner B, et al. Polyploids exhibit higher potassium uptake and salinity tolerance in Arabidopsis. Science. 2013;341(6146):658-9.
64. Wu G-Q, Lin L-Y, Li S-J. Tetraploid exhibits more tolerant to salinity than diploid in sugar beet (*Beta vulgaris* L.). Acta Physiol Plant. 2019;41:52.
65. Meng H-B, Jiang S-S, Hua S-J, Lin X-Y, Li Y-L, Guo W-L, et al. Comparison Between a Tetraploid Turnip and Its Diploid Progenitor (*Brassica rapa* L.): The Adaptation to Salinity Stress. Agricultural Sciences in China. 2011;10:363-75.
66. Xue H, Zhang F, Zhang Z-H, Fu J-F, Wang F, Zhang B, et al. Differences in salt tolerance between diploid and autotetraploid apple seedlings exposed to salt stress. Scientia Horticulturae. 2015;190:24-30.
67. Tu Y, Jiang A, Gan L, Hossain M, Zhang J, Peng B, et al. Genome duplication improves rice root resistance to salt stress. Rice. 2014;7:15.
68. Jiang M, Xu F, Peng M, Huang F, Meng F. Methyl jasmonate regulated diploid and tetraploid black locust (*Robinia pseudoacacia* L.) tolerance to salt stress. Acta Physiol Plant. 2016;38:106.
69. Tal M, Gardi I. Physiology of Polyploid Plants: Water Balance in Autotetraploid and Diploid Tomato under Low and High Salinity. Physiol Plant. 1976;38:257-61.
70. Taleisnik E, Peyrano G, Arias C. Response of *Chloris gayana* cultivars to salinity. 1. Germination and early vegetative growth. Tropical Grasslands. 1997;31:232-40.
71. Mouhaya W, Allario T, Brumos J, Andrés F, Froelicher Y, Luro F, et al. Sensitivity to high salinity in tetraploid citrus seedlings increases with water availability and correlates with expression of candidate genes. Funct Plant Biol. 2010;37:674-85.
72. Garbutt K, Bazzaz FA. Leaf demography, flower production and biomass of diploid and tetraploid populations of Phlox drummondii Hook. on a soil moisture gradient. New Phytol. 1983;93:129-41.
73. Tyler B, Borrill M, Chorlton K. Studies in *Festuca*. X. Observations on Germination and Seedling Cold Tolerance in Diploid *Festuca pratensis* and Tetraploid *F. pratensis* var. *Apennina* in Relation to Their Altitudinal Distribution. Journal of Applied Ecology. 1978;15:219-26.
74. Sugiyama S. Differentiation in competitive ability and cold tolerance between diploid and tetraploid cultivars in Lolium perenne. Euphytica. 1998;103:55-9.
75. Thompson KA, Husband BC, Maherali H. Climatic niche differences between diploid and tetraploid cytotypes of Chamerion angustifolium (Onagraceae). Am J Bot. 2014;101(11):1868-75.
76. Lu H, Xue L, Cheng J, Yang X, Xie H, Song X, et al. Polyploidization-driven differentiation of freezing tolerance in Solidago canadensis. Plant Cell Environ. 2020;43(6):1394-403.
77. Zhang XY, Hu CG, Yao JL. Tetraploidization of diploid Dioscorea results in activation of the antioxidant defense system and increased heat tolerance. J Plant Physiol. 2010;167(2):88-94.

78. Li WD, Hu X, Liu JK, Jiang GM, Li O, Xing D. Chromosome doubling can increase heat tolerance in *Lonicera japonica* as indicated by chlorophyll fluorescence imaging. Biologia Plantarum. 2011;55:279-84.
79. Niwa Y, Sasaki Y. Plant self-defense mechanisms against oxidative injury and protection of the forest by planting trees of triploids and tetraploids. Ecotoxicology and Environmental Safety. 2003;55:70-81.
80. Chandra A, Dubey A. Effect of ploidy levels on the activities of delta(1)-pyrroline-5-carboxylate synthetase, superoxide dismutase and peroxidase in Cenchrus species grown under water stress. Plant Physiol Biochem. 2010;48(1):27-34.
81. Gupta PP. Consequences of artificial and natural chromosome doubling on DNA, RNA and protein contents in *Cochlearia* (Brassicaceae). Plant Syst Evol. 1981;138:23-7.
82. Dai F, Wang Z, Luo G, Tang C. Phenotypic and Transcriptomic Analyses of Autotetraploid and Diploid Mulberry (Morus alba L.). Int J Mol Sci. 2015;16(9):22938-56.
83. Godfree RC, Marshall DJ, Young AG, Miller CH, Mathews S. Empirical evidence of fixed and homeostatic patterns of polyploid advantage in a keystone grass exposed to drought and heat stress. R Soc Open Sci. 2017;4(11):170934.
84. Van Drunen WE, Husband BC. Immediate vs. evolutionary consequences of polyploidy on clonal reproduction in an autopolyploid plant. Ann Bot. 2018;122(1):195-205.
85. Ma Y, Xue H, Zhang L, Zhang F, Ou C, Wang F, et al. Involvement of Auxin and Brassinosteroid in Dwarfism of Autotetraploid Apple (Malus x domestica). Sci Rep. 2016;6:26719.
86. Tan FQ, Tu H, Liang WJ, Long JM, Wu XM, Zhang HY, et al. Comparative metabolic and transcriptional analysis of a doubled diploid and its diploid citrus rootstock (C. junos cv. Ziyang xiangcheng) suggests its potential value for stress resistance improvement. BMC Plant Biol. 2015;15:89.
